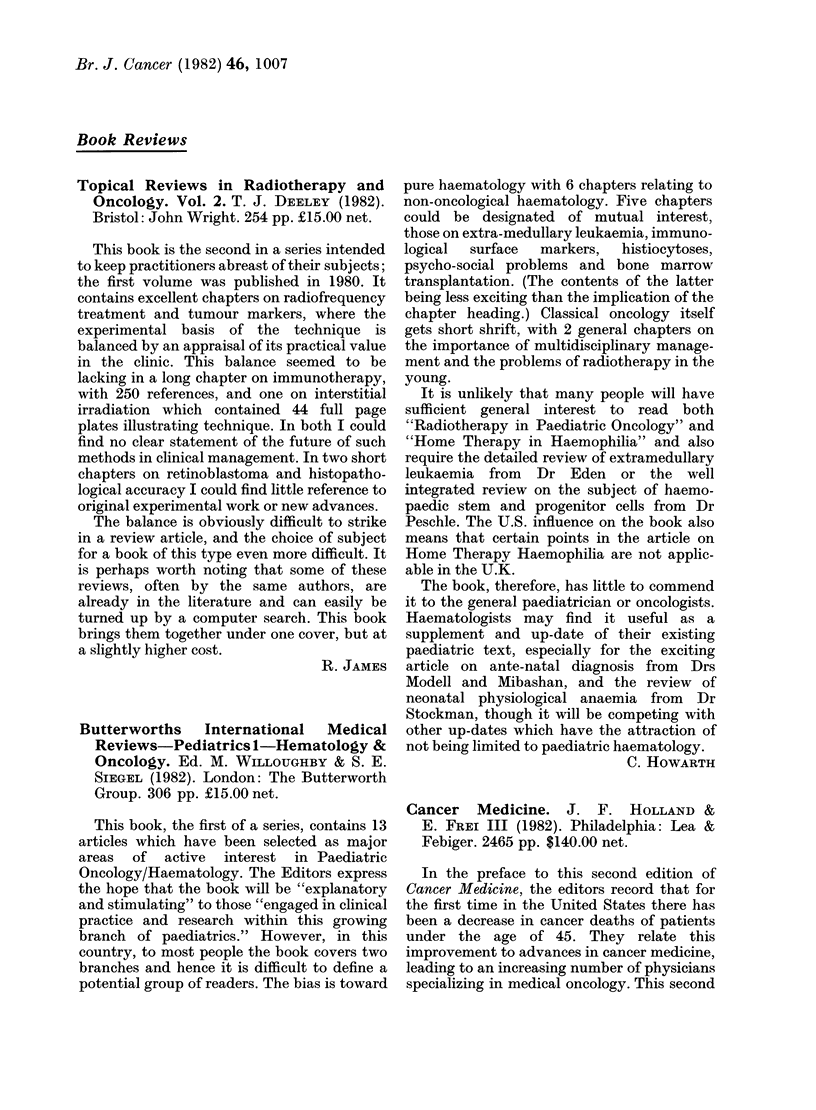# Topical Reviews in Radiotherapy and Oncology. Vol. 2

**Published:** 1982-12

**Authors:** R. James


### *Book Reviews*

**Topical Reviews in Radiotherapy and Oncology. Vol. 2.** T. J. DEELEY (1982). Bristol: John Wright. 254 pp. £15.00 net.

This book is the second in a series intended to keep practitioners abreast of their subjects; the first volume was published in 1980. It contains excellent chapters on radiofrequency treatment and tumour markers, where the experimental basis of the technique is balanced by an appraisal of its practical value in the clinic. This balance seemed to be lacking in a long chapter on immunotherapy, with 250 references, and one on interstitial irradiation which contained 44 full page plates illustrating technique. In both I could find no clear statement of the future of such methods in clinical management. In two short chapters on retinoblastoma and histopathological accuracy I could find little reference to original experimental work or new advances.

The balance is obviously difficult to strike in a review article, and the choice of subject for a book of this type even more difficult. It is perhaps worth noting that some of these reviews, often by the same authors, are already in the literature and can easily be turned up by a computer search. This book brings them together under one cover, but at a slightly higher cost.

R. JAMES